# MetaZipf. A dynamic meta-analysis of city size distributions

**DOI:** 10.1371/journal.pone.0183919

**Published:** 2017-08-29

**Authors:** Clémentine Cottineau

**Affiliations:** Urban Dynamics Lab, Centre for Advanced Spatial Analysis, University College London, W1T 4TJ, United Kingdom; East China University of Science and Technology, CHINA

## Abstract

The results from urban scaling in recent years have held the promise of increased efficiency to the societies who could actively control the distribution of their cities’ size. However, little evidence exists as to the factors which influence the level of urban unevenness, as expressed by the slope of the rank-size distribution, partly because the diversity of results found in the literature follows the heterogeneity of analysis specifications. In this study, I set up a meta-analysis of Zipf’s law which accounts for technical as well as topical factors of variations of Zipf’s coefficient. I found 86 studies publishing at least one empirical estimation of this coefficient and recorded their metadata into an open database. I regressed the 1962 corresponding estimates with variables describing the study and the estimation process as well as socio-demographic variables describing the territory under enquiry. A dynamic meta-analysis was also performed to look for factors of evolution of city size unevenness. The results of the most interesting models are presented in the article, whereas all analyses can be reproduced on a dedicated online platform. The results show that on average, 40% of the variation of Zipf’s coefficients is due to the technical choices. The main other variables associated with distinct evolutions are linked to the urbanisation process rather than the process of economic development and population growth. Finally, no evidence was found to support the effectiveness of past planning actions in modifying this urban feature.

## Introduction

The regularity in city size distribution has been known for more than a century [1]. Auerbach found that the multiplication of cities’ size (population) by their rank (by decreasing population) resulted in a constant quantity. The rank-size power law was then made famous by G. Zipf [2] when he stated that this constant should be 1, kick-starting a long-standing fascination as well as a stream of research to explain this “mysterious” precision [3].

More recently, research on urban scaling laws has shown that some indicators of urban efficiency could be expressed as a power law of urban population [4, 5]. For example, total income and GDP generally tend to scale superlinearly, i.e. the aggregate output in a city *i* of population double to that of *j* is more than twice the output of *j*. In other words, larger cities tend to be, on average, richer *per capita*. On the contrary, the amount of infrastructure scales sublinearly and shows economies of agglomeration in larger cities. The resulting aggregate amount of wealth or infrastructure in a system thus depends eventually on the distribution of sizes of the different cities in the system (even though the mechanism behind scaling operates within the cities themselves). These urban features can be linked back to the size distribution of cities in a given system [6]. This link between Zipf’s law and urban scaling have lead some authors to suggest promising opportunities for urban planning: to maximise efficiency, it could be sufficient to tweak the slope of the rank-size distribution! (Note that in this article as in the literature in general, the terms “rank-size rule”, “Zipf’s law” and “city size distribution” are used as synonyms).

However, before trying to inform policies in such a way, three questions arise: 1/ is it possible to control the distribution of city sizes in a country at all? 2/ What drives the value of this slope? and 3/ have previous policies ever been successful in modifying the slope of the rank-size rule? I will try to answer these questions, relying on the past 100 years of literature, starting by unpicking undesirable fluctuations from genuine variations in the measured values of urban unevenness. The deviations observed thus can arise from four sources: estimation biases, measurement “errors”, authentic spatio-temporal variations and as a result of planning policies.

### Estimation biases

There are two ways of estimating the rank-size rule. In the tradition of Lotka [7], the population of cities is expressed as a power law of their rank (Eq 1). The regression formula for the empirical fit is then expressed as in Eq 2.

$$\begin{array}{c} P_{i}=\beta *{R_{i}}^{\alpha }. \end{array}$$ (1)

$$\begin{array}{c} \text{ln}\left(P_{i}\right)=b+\alpha *ln\left(R_{i}\right)+e_{i} \end{array}$$ (2)

with: *P*_*i*_ the population of city i, *R*_*i*_ its rank in the ordered distribution of populations, *e*_*i*_ the randomly distributed error term and *b* = *ln*(*β*).

In the economic tradition, authors have favoured the Pareto form, which expresses the rank of cities as a power law of their population (Eq 5). The regression formula for the empirical fit is then expressed as in Eq 4.

$$\begin{array}{c} R_{i}=\beta^{\prime }*{P_{i}}^{\alpha^{\prime }} \end{array}$$ (3)

$$\begin{array}{c} \text{ln}\left(R_{i}\right)=b^{\prime }+\alpha^{\prime }*ln\left(P_{i}\right)+{e_{i}}^{\prime } \end{array}$$ (4)

with: *α*′ = 1/*α* and *b*′ = *ln*(*β*′).

The two forms should give equivalent results after transformation of the exponent *α* [8]. This paper shows that this is not necessarily the case. The second possible source of bias in the estimation of *α* is the way the regression equation is fitted to the data. Until recently, all estimations were produced using the Ordinary Least Squares (OLS) method for the linear regression of logged populations and ranks. Gabaix and Ibragimov [9] found this method to be faulty when applied to small samples of cities. They proposed a simple fix: they subtract 1/2 to the rank value. The OLS method itself is criticised and has been replaced by the Maximum Likelihood Estimation or by Markov Chains methods in recent studies. Regardless of the precision of the estimation procedure, the data used to fit the model can still be a source of bias and variation.

### Measurement “errors”

Measurement “errors”, or more generally differences in *α* estimates which are due to data collection choices, can be summarised into two categories. The first one refers to the definition of cities for which population is collected. Indeed, because the concept of city is broad, there are various delineations which can be used as units *i* in the rank-size regression. For example, cities can be considered as local administrative entities such as municipalities, as morphological units defined by the continuity of the built environment or the connectivity of street intersections [10], or as metropolitan areas defined with respect to the integration of commuting flows. Because the latter is an aggregation of local units, choosing one or the other definition will affect the number of cities considered and their individual population, thus potentially impacting the value of *α* for the same country at the same date. The same holds true when local government reforms imply substantial mergers of municipalities [11].

The minimum population criterion for inclusion of settled units into the set is the second choice which affects the estimation of the rank-size rule, especially when the logged rank-size curve is not completely straight but somehow convex or concave, which is frequent in empirical systems [12]. Consequently, these biases have to be accounted for in order to evaluate the ‘genuine’ effect of spatio-temporal features.

### Spatio-temporal variations

Deviations to the “law” have been understood either as proofs against the validity of the law, either as indications of urban particularities. For example, Moriconi-Ebrard et Pumain [13, 14] have suggested that the speed of transportation systems at the time of urbanisation could have influenced the hierarchy of city sizes, because in recently urbanised systems long distance could be reached more easily, court-circuiting the need for a large number of small cities between large settlements. Consequently, the rank-size curve should be steeper and the value of *α* higher.

Alternatively, Morrill [15] and Rosen & Resnick [16] have suggested that small territories would be more uneven than large ones because the concentration of power in the largest cities in the latter would not be balanced by a sufficiently large set of secondary cities. In the same range of ideas, loosely-integrated urban systems have been said to be more prone to deviate from Zipf’s law than integrated ones because they would duplicate small to medium-size cities and/or lack a primate city [16–18].

Finally, and most interesting for my purpose here, systems of cities have been said to tend towards more unevenness of time [8, 11, 14], notably because larger cities tend to grow faster on average, being first-movers on innovation creation and adoption [14].

### Purpose of the study

Given these insights, is it possible to control for measurement errors and estimation biases to reveal the “true” effect of time, development and demographic growth on the evolution of *α*? Instead of estimating yet another set of *α* from empirical data, I choose to build on the accumulated literature and to answer this question with a meta-analysis of the rank-size estimates published in previous studies. A meta-analysis of Zipf’s law exists already [8], but it is restricted to evaluating the technical biases of estimation rather than looking for territorial drivers of deviations. In this contribution:

- I add more papers to the analysis (published since Nitsch’s article in 2005 for instance), especially from the non-economics literature;
- I add more variables in the meta-regression to find spatio-temporal drivers of city size unevenness;
- I control for the regression method (OLS, Maximum Likelihood, etc.);
- I distribute the resulting database online under an open-licence: https://github.com/ClementineCttn/MetaZipf and along with a dedicated website which allows to reproduce the analysis: https://clementinegeo.shinyapps.io/MetaZipf/.

These significant additions lead to new insights regarding the dynamics of Zipf’s law and the potentials for urban planning in this context.

## Materials and methods

### Metadata as data

In this section, I explain the selection of studies to be included in the meta-analysis, as well as the way the data was collected and formatted to test my hypotheses. I then give an overview of a century of publication of Zipf’s estimates.

#### Data collection

An important aspect of meta-analyses has to do with the selection of the studies to be included in the analysis. The selection criteria should be broad enough to allow a large number of studies to be considered, yet systematic enough to be reproducible.

In a previous meta-analysis of Zipf’s estimates, V. Nitsch [8] used the search words “rank AND size AND cities” in the EconLit database. He then filtered out the non-empirical studies and added some references which were absent from the EconLit results but present in the bibliographies of many studies found that way. He ended up with a list of 29 references. Although the first selection criterion is systematic, the largest flaw with this approach is that it introduces a very large bias towards studies published in economics journals, ignoring the contributions from social sciences which are not included in the EconLit database. Furthermore, the second round of selection appears non systematic. Finally, the resulting database used for the meta-analysis is not available to the reader.

In this study, I take advantage of new technologies of interactive online tools and crowdsourcing to establish a pool of studies as exhaustive as possible in relation to Zipf’s law. All studies are included in the meta-analysis as long as:

1. They contain at least one estimate of the rank-size exponent based on population
2. The regression is made on empirical urban data
3. The regression model is bivariate (i.e. relating populations and ranks or ranks—1/2, but not to any other instrumental variable).

At the time of writing, I had collected 86 such studies, and for each of them, recorded the series of specifications which characterises the estimation of Zipf’s law and could influence its value. The analysis is reproducible with this selection, although the database will continue to grow.

**Fig 1:**
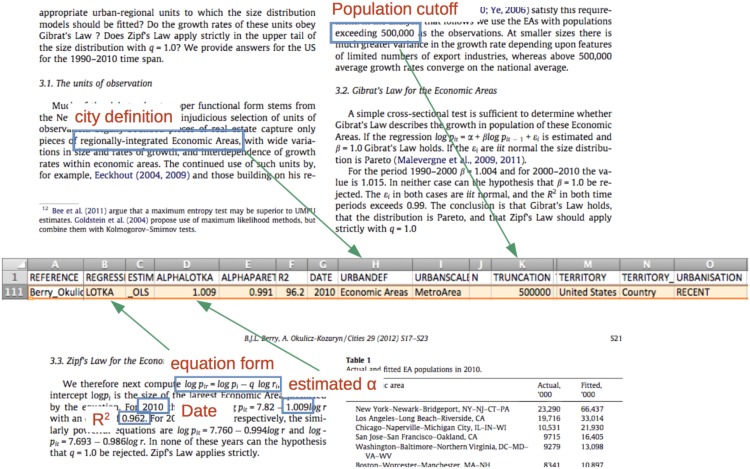
Metadata as data. The information relating to the estimation of the slope of the rank-size curve in the study is recorded and formatted in a consistent way to serve as data in the meta-analysis.

As shown in Fig 1, the data collection involves reading through the paper and recording the relevant pieces of information into a consistent and machine-readable format. Put together, the 86 studies give 1962 estimates of the rank-size coefficient, which correspond to 1962 different regressions and their specifications.The meta-elements associated with each value of estimate are the following:

- REFERENCE: the reference ID, which links an estimate to the study where it was published. Each study contains from 1 to 255 empirical estimates.
- ALPHA: the value of the estimate. Some were produced using the Lotka form (Eq 1) and other using the Pareto form (Eq 5). In any case, the value for the other form can be easily computed as *α*′ = 1/*α*. In the present paper, I choose to express all results using the Lotka form, thus interpreting the coefficient *α* as an index of unevenness of size in a given urban system.
- *R*^2^: the r-squared value of the regression. The information was available only half of the time.
- ESTIMATION: the method used to fit the regression model. OLS refers to the Ordinary Least Squares method, GI to Gabaix and Ibragimov’s trick of using rank—1/2, MC stands for Markov Chains and ML for Maximum Likelihood estimations.
- DATE: the year to which the population of cities refer (which is different from the publication year). The distribution of these dates goes from 1600 to 2014.
- TERRITORY: the administrative boundaries within which cities are drawn.
- URBANDEF: the way cities are defined in the study, using the original and heterogenous phrasing of the authors.
- N: the number of cities used in the regression. One third of that information is not reported.
- TRUNCATION: the minimum population of cities used in the regression. A quarter of that information is not reported.

In addition to the specifications of the estimations, which can vary within a single study, I recorded some particularities of the study itself, in the same way as in [8]. I used the year of publication, the number of estimates published alongside the one under enquiry, as well as the period covered and the number of territories investigated.

#### Additional data

Because of the heterogeneity of the data and the way it is reported, an additional variable was constructed. URBANSCALE is the simplification of the URBANDEF categories. It has only four values which correspond to a conceptual way of defining cities. It takes the value “LocalUnits” when the URBANDEF corresponds to municipalities, communes or places, i.e. administrative way of delineating cities. It takes the value “MorphoCities” for definitions using the continuity of the built environment as a criterion to define cities (urban areas or urban agglomerations for example). It takes the value “MetroArea” when a functional criterion is used to define cities (MSA in the Unites States, FUA in Europe for example). It takes the value “VariaMixed” in all other cases.

Finally, and this is how this new meta-analysis innovates, four topical variables were added to the technical variables classically used. For estimates obtained within national boundaries, I matched the meta-database with historical datasets from the United Nations and the World Bank to add the total population [19], GDP per capita [20] and urbanisation level (% of the population which is urban) [21] at the date corresponding to the data from the study. I also added a binary variable describing the age of the urbanisation. It is set as “old” in areas that were urbanised more than a millennium ago: in Europe, Asia and the Middle East. It is set as “recent” for the American, Oceanian and African continents. The dichotomy is pretty crude but has the merit of enabling to test the hypothesis according to which ‘old urban systems’ are more even in terms of city sizes.

#### Overview of a century of publication

**Fig 2:**
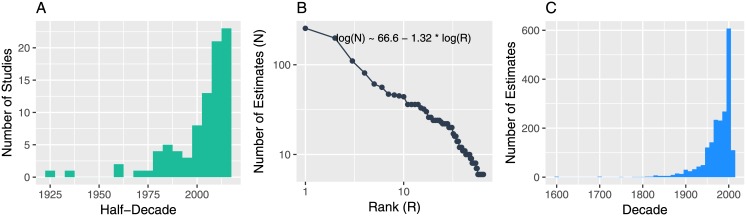
Temporal overview of the metadata. A: Distribution of studies publishing Zipf’s estimates over time. B: rank size distribution of the number of estimates per study. C: Distribution of estimates of Zipf by year of the data used in the estimation.

The resulting database is composed of 1962 estimates covering all the continents and spanning over 400 years. They come from 86 studies published in a variety of journals (the highest provider being Urban Studies with 8 studies and the Journal of Regional Science with 6). There seems to have been a fashion in publishing empirical estimates over the last two decades, with 54 of the 86 studies included published since 2000, although I detect a peak in publication about empirical estimations of Zipf’s law in the 1980s too (Fig 2A). Consequently, estimations relating to the year 2000 or 2001 are dominant in the set (Fig 2C), and in the majority of cases, estimates refer to urban data collected after the 1950s. The distribution of estimates by studies is itself skewed and if I estimate the relation between its rank and its size with the Lotka form (Fig 2B), I find a coefficient of 1.32, i.e. showing more unevenness than in city systems! Indeed, a handful of authors have published a large mass of estimates whereas most of them only published a handful of estimates in each study.

The studies with the most estimates published are comparative studies with measures for many countries in the world (255 estimates in [13], 99 in [22]; 61 in [23], 56 in [16]). Another type of studies with a large number of estimates corresponds to sensitivity analyses on a single territory, with variations of dates and cutoff values (110 estimates in the United States in [24], 45 estimates in China in [25]). Those studies are over-represented in the meta-analysis when each estimate counts as one observation. However, they have the advantage of providing comparable cases to better isolate the effect of unique factors (minimum population, year, etc.), everything else being equal. I therefore weight estimates equally, irrespective of the study in which they were published. However, as in Nitsch’s study, I control for study effects with fixed-effects and panel models.

**Fig 3:**
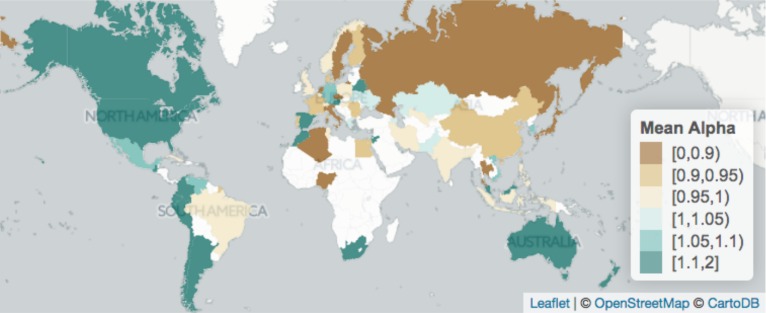
Geographical overview of the metadata. Mean value of alpha reported by country in the literature reviewed.

In terms of geographical coverage, the picture is one of large urban unevenness in the world. Some visual trends arise from Fig 3: American and Australasian cities seem more unevenly distributed in each countries, whereas Asian cities seem more evenly distributed in terms of size. The trend in European and African countries is much less clear-cut.

### Static and dynamic meta-analyses

To conduct the meta-analysis, I related the values of *α* estimates with the characteristics of the estimations, studies and territories at the time of observation. There are various non-equivalent ways to do this meta-analysis.

#### Ordinary linear models

For the static analysis, I use ordinary bivariate then multivariate linear models (Eq 5).

$$\begin{array}{c} \alpha_{i,j,t}=b+c*X_{i,j,t}+\epsilon \end{array}$$ (5)

with: *X*_*i*,*j*,*t*_ a vector of variables describing the estimate *α* in the observation *i* in study *j* at time *t*, and *ϵ* a randomly distributed error.

In the bivariate models, the correlation between a single variable and the value of *α* is estimated. In multivariate models, I draw variables from three sets representing different regimes of explanation (Table 1):

1. Estimation variables refer to the way the estimation *i* is performed.
2. Study variables refer to the attributes of the publication *j* where *i* was reported.
3. Territory variables refer to the attributes of the urban system and its territorial envelope at the time *t* of its observation.

The first two sets correspond to the technical drivers of variation whereas variables of the territorial set are used to test theoretical hypotheses of change in the urban hierarchy as measured by Zipf’s law.

**Table 1:** Three sets of static variables.

Estimation variables (i)	Study variables (j)	Territory variables (t)
City definition	Year of publication	Date
Population Cutoff	Number of Estimates	Total population
Number of cities	Period covered	GDP per capita
Estimation method	Number of countries	Age of urbanisation
Regression form	-	Urbanisation level

In order to control for biases such as correlated errors within the same publication, I also present results estimated with a fixed-study effects model where I simply add an error term *u*_*j*_ to Eq 5.

#### Dynamic meta-analysis

The models for a static meta-analysis can include the date of observation, but they are not really suited for looking at dynamic and longitudinal evolutions. For that purpose, I use two strategies. Firstly, I regress the static value of *α* with a random effect panel model (Eq 6). This method helps controlling for correlated errors within units and over time.

$$\begin{array}{c} \alpha_{i,j,t}=b+c*X_{i,j,t}+U_{i,j,t}+\epsilon_{i,j,t} \end{array}$$ (6)

with: *U*_*i*,*j*,*t*_ the between-entity error and *ϵ*_*i*,*j*,*t*_ the within-entity error.

Secondly, I then compute the average annual growth rate of *α* values estimated under the same specifications with three sets of variables (Table 2):

1. Territory variables refer to the attributes of the urban system and its territorial envelope at the time *t*_1_ of initial observation.
2. Dynamic variables refer to the growth of territorial variables between *t*_1_ and *t*_2_.
3. Event variables refer the presence or absence of territorial events between *t*_1_ and *t*_2_.

**Table 2:** Three sets of dynamic variables.

Territory variables (*t*_1_)	Dynamic variables (*t*_1_-*t*_2_)	Event variables (*t*_1_-*t*_2_)
Initial value of *α*	Population growth	Revolution
Initial population	GDP per capita growth	War of Independence
Initial GDP per capita	Urbanisation level growth	Civil War
Initial Urbanisation level	Number of cities growth	International War

All these models were estimated using R and all their results are reproducible on the MetaZipf application, which is free and open: https://clementinegeo.shinyapps.io/MetaZipf/.

## Results

**Fig 4:**
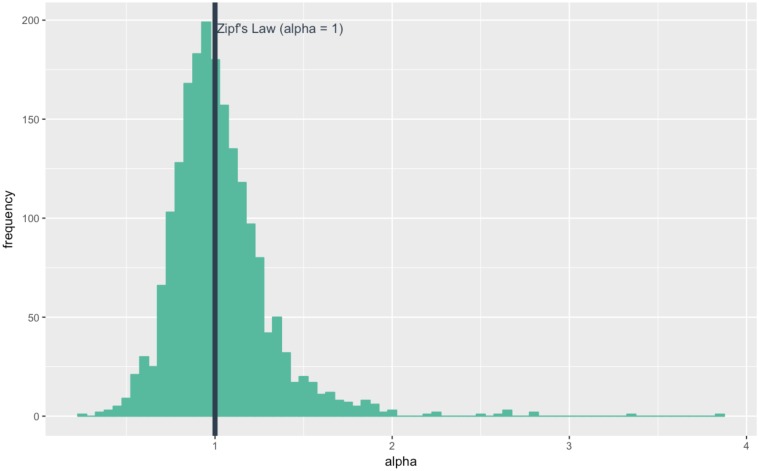
Distribution of *α* values: 1962 estimates reported in the literature reviewed.

The overall distribution of *α* values estimated in the literature and reviewed in this paper is rather symmetrical and centred on 1, as the mean value is 1.025 and the median 0.986 (Fig 4). This aggregate result somehow confirms the fascinating regularity of Zipf’s law. It updates and contradicts the mean value found by Nitsch [8] on 29 studies, which he found to be significantly different from 1. However, I too find that the mode of the distribution indicates that urban systems slightly more even than expected from Zipf’s law. The standard deviation (0.282) is however important for a power law exponent, which justifies the search for explaining factors. If these deviations were only attributable to differences in methodology, the regularity would outweigh the irregularity, and that policy conclusions could only be weak. If not, the urban hierarchy measured by the rank-size rule would vary with systematic factors, and so policies could have some leverage on the inequality level of city sizes.

### Bivariate static relationships

It turns out that all bivariate linear models are statistically significant, although none of them reaches an *R*^2^ of more than 5%. In general, the variables relating to estimation variables have slightly higher explaining powers, whereas study variables have the lowest *R*^2^ (Table 3).

**Table 3:** Descriptive statistics and bivariate regressions.

	Descriptive Statistics			Bivariate meta-regression				
	Mean	Std. dev.	n	p-value	Intercept	Coefficient	t value	*R*^2^ (%)
All estimates	1.025	0.282	1962					
*Estimation characteristics*								
**City Definition:** LocalUnit	1.018	0.291	1209	0.000	1.018		127.152	2.27
MorphoCity	1.075	0.280	516			*0.000*	0.017	
MetroArea	1.019	0.137	139			0.057	3.885	
VariaMixed	0.855	0.255	98			-0.163	-5.584	
**Population cutoff:** 10,000 or lower	1.016	0.239	289	0.000	1.016		112.965	3.73
] 10,000; 100,000 [	1.051	0.359	938			*0.035*	1.879	
100,000 or higher	0.889	0.200	208			-0.128	-6.017	
Missing specification	1.082	0.313	517			0.066	4.348	
**Number of Cities:** 30 or lower	1.052	0.311	171	0.000	1.052		49.797	4.27
] 30; 300 [	0.947	0.276	681			-0.105	-4.425	
300 or higher	1.047	0.277	382			*-0.005*	-0.194	
Missing specification	1.081	0.268	728			*0.029*	1.219	
**Estimation Method:** OLS	1.026	0.250	1603	0.000	1.026		146.381	1.19
Gabaix & Ibragimov	0.957	0.252	197			-0.069	-3.254	
Markov Chains	1.142	0.235	10			*0.116*	1.301	
Maximum Likelihood	1.098	0.522	152			0.072	3.025	
**Regression form:** Lotka	0.987	0.201	737	0.000	0.987		95.507	1.09
Pareto	1.048	0.319	1225			0.061	4.653	
*Study characteristics*								
**Year of Publication:** 1975 or before	0.981	0.281	42	0.000	0.981		22.652	1.05
] 1975; 2000 [	0.982	0.168	555			*0.000*	0.007	
2000 or after	1.044	0.315	1365			*0.063*	1.432	
**Number of estimates:** 1 only	0.957	0.110	11	0.006	0.957		11.280	0.53
] 1; 10 [	0.959	0.220	160			*0.002*	0.021	
10 or more	1.032	0.287	1791			*0.074*	0.872	
**Period covered:** 1 year	0.927	0.231	160	0.000	0.927		41.779	1.08
] 1; 50 [	1.032	0.242	1120			0.105	4.423	
50 years or more	1.037	0.344	682			0.110	4.456	
**Number of countries:** 1 only	1.062	0.289	687	0.000	1.062		99.164	1.14
] 1; 5 [	0.971	0.371	249			-0.091	-4.381	
5 or more	1.014	0.248	1026			-0.048	-3.443	
*Territorial characteristics*								
**Date of observation:** 1940 or before	0.963	0.242	245	0.000	0.963		53.609	0.70
] 1940; 2000 [	1.032	0.287	1189			0.069	3.502	
2000 or after	1.038	0.285	528			0.075	3.433	
**Total Population:** 10 million or less	1.024	0.190	230	0.000	1.024		55.170	0.58
] 10M; 100M [	1.059	0.236	552			*0.035*	1.562	
100 million or more	1.014	0.348	492			*-0.010*	-0.453	
Missing data	1.007	0.287	688			*-0.017*	-0.788	
**GDP per Capita:** 1000 Current USD or less	0.976	0.285	317	0.000	0.976		61.917	1.23
] 1,000; 10,000 [	1.049	0.236	443			0.073	3.557	
10,000 or more	1.073	0.306	327			0.098	4.409	
Missing data	1.013	0.289	875			0.037	2.034	
**Age of urbanisation:** Old	0.993	0.286	1301	0.006	0.993		129.022	3.28
Recent	1.094	0.261	649			0.102	7.611	
Not applicable	0.792	0.141	12			-0.201	-2.492	
**Urbanisation rate:** 20% or less	1.101	0.575	72	0.000	1.101		33.325	1.45
] 20; 60 [	0.992	0.214	553			-0.109	-3.100	
60% or more	1.065	0.273	649			*-0.036*	-1.037	
Missing data	1.007	0.287	688			-0.094	-2.695	

#### Estimation variables

Although city definitions have various frequencies (1209 out of 1962 values of *α* were estimated using local units as “cities”), there is evidence that built-up areas (or *MorphoCities*) are more uneven on average than local units. This pattern has been observed empirically when comparing the two definitions within the same territory [1, 16, 22]. The reason for this is that morphological agglomerations are aggregates of local units and than on average, large built-up areas concentrate more units than small areas where the two definitions match. While comparing ‘natural’ cities defined as agglomeration of street intersections and urban areas in the United States, [10] found that more than a simple difference in values of *α*, the choice of city definition also determines if the model is robust at all or not.

The population cutoff used to select cities is an important factor affecting the value of Zipf’s estimate. Unfortunately, in 25% of all cases, the value of this cutoff is not reported in the publication. Lower cutoffs tend to be associated with larger inequality of city sizes, and larger cutoffs with more evenness. This is linked to the fact that rank-size curves are not always perfectly straight (i.e. not truly scale-free [10]) but show some convexity [12, 13, 16, 22]. Observing a small set of large cities thus leads to underestimate artificially the index of urban hierarchy.

The number of cities used in the regression is somehow dependent on the cutoff, but also on the size of the country. It is also massively under-reported (728 missing values). It shows a suspiciously high average *α* for data sets of less than 30 observations, which can be regarded as too small and inconsistent to be interpreted [16, 26]. The extreme values I recorded all correspond to these very small sets. However, sets of 30 to 300 cities show a low degree of inequality, which matches the previous observation: the top of the urban distribution is more even than the whole set. This has been interpreted as a lack of integration in large systems [17, 18].

A factor that has not been tested systematically so far is the impact of the estimation method on the value of the estimate, although the vast majority of published results were obtained using OLS. I find a significantly lower average value of *α* when authors have used the method of Gabaix and Ibragimov [9]. Additionally, I find a significant and worrying effect of the regression form used, with Pareto estimations giving more uneven results on average.

#### Study variables

Using variables describing the study where estimates were published, I find that:

- Recent studies tend to publish, on average, higher values of *α* (1.04), whereas studies published before 2000 have average estimates of 0.98 (unlike [8]).
- Studies reporting more than 10 estimates tend also to report, on average, higher values of *α* (1.03), whereas studies with less than 10 estimates have an average value of 0.96 (like [8]).
- Studies reporting estimates for more than one date tend also to report, on average, higher values of *α* (1.03), whereas studies for one year only have an average value of 0.93 (like [8]).
- Studies reporting estimates for 2 to 4 countries tend also to report, on average, lower values of *α* (0.97), whereas studies for one country only (1.06) or more than five of them (1.01) report higher unevenness (like [8]).

#### Territory variables

Finally, my interest lies in the last set of variables describing urban systems and their territorial envelope.

In terms of evolution, I find that estimates for city systems of 1940 and before are reported with significantly lower values on average compared to estimates for cities in the 1940s to 1990s. This result could be interpreted in two ways, which this simple model cannot disentangle. Firstly, this could confirm theories about the tendency of city systems to grow more uneven with time [8, 14]. Second, it could correspond to the different mix of countries studied over time, as developed countries of the western world (and their colonial empire) form the vast majority of available data for periods prior to 1940. Therefore, the higher average unevenness might not be a consequence their own evolution but could result from the addition to the literature of case studies from the developing world.

Although some scholars have argued that cities of smaller countries should be more unevenly distributed because powers are relatively more concentrated in the primate city [15, 16], I find no evidence of such relationship: estimates from large countries (≥ 100 million inhabitants) are on average very similar to the estimates in small countries (≤ 10 million), although a third of data are missing.

Richer countries tend to be associated with more unevenness in city sizes, although the information is missing in 875 cases, and the *R*^2^ is low. Nonetheless, the relationship is ordered systematically, with countries with less than 1,000 current US dollars per capita having an average value of *α* equal to 0.976, whereas the average is 1.049 for intermediate countries and 1.073 for countries with more than 10,000 dollars per capita.

Cities in territories urbanised early on appear more evenly distributed in terms of size (*α* = 0.993), compared to newly urbanised systems (*α* = 1.094). One explanation from Evolutionary Urban Geography [13, 14] is that because the transport networks available at the time were slower than today, a larger amount of small cities were necessary. In areas urbanised with railways and highways, these small cities tend to have been short-circuited, leading to more uneven distribution of sizes.

Finally, city systems are more uneven when urbanisation is still low (≤ 20% of the national population lives in a UN defined city) compared to intermediate levels of urban population (from 20 to 60%). Although not significantly, this value tends to increase again above 60% suggesting a phenomenon opposite to Kuznets’ curve of income inequality. According to Kuznets [27], countries start their economic development with a low level of income inequality. The rising income due to development is at first unevenly distributed among citizens, but this inequality then tends to lessen as progress increases everyone’s situation. In the case of urban inequality, I find that countries start the urban transition from a situation where there is high unevenness between city sizes (usually one large city and a few emerging towns). Rural migrations and urban growth then pushed second-tier cities to even the distribution of city size, whereas saturated territories encounter a new rise of Zipf’s coefficient. Metropolises keep growing in interaction with other world cities whereas small cities stagnate or shrink [28].

### Standard meta-analysis and the importance of technical specifications

Running a first meta-analysis on the full set of variables, I obtain a model representing 19.3% of the total variance in *α* values (Table 4). All variables are significantly associated with a variation of *α*, except the time coverage of the study, the population and GDP per capita of corresponding countries. In terms of territorial variables, I find that city systems before 1940 appear systematically more even, and so do systems where the urbanisation level is intermediate. Finally, I confirm the higher unevenness of systems in recently urbanised territories. This way of introducing territorial variables in the meta-analysis is novel and suggests that the inequality of city sizes has more to do with the urbanisation process itself rather than economic and demographic development. However at this stage, the model is too coarse to disentangle technical specifications from theoretical drivers.

**Table 4:** OLS meta-analysis. Complete linear model regressing *α* value with estimation, study and territorial variables.

	Coefficient	Std. error	t-value	p-value
Intercept	0.607	0.082	7.392	0.000
*Estimation characteristics*				
**City Definition:** LocalUnit	*reference category*			
MorphoCity	0.157	0.018	8.745	0.000
MetroArea	*-0.04*	*0.03*	*-1.53*	*0.12*
VariaMixed	-0.177	0.030	-5.845	0.000
**Population cutoff:** 10,000 or lower	*0.02*	*0.02*	*1.29*	*0.20*
] 10,000; 100,000 [	*reference category*			
100,000 or higher	-	-	-	-
Missing specification	0.119	0.018	6.611	0.000
**Number of Cities:** 30 or lower	*-0.02*	*0.01*	*-1.04*	*0.30*
] 30; 300 [	*reference category*			
300 or higher	-	-	-	-
Missing specification	0.094	0.017	5.513	0.000
**Estimation Method:** OLS	*reference category*			
Gabaix & Ibragimov	*-0.03*	*0.02*	*-1.34*	*0.18*
Markov Chains	*0.02*	*0.08*	*0.19*	*0.85*
Maximum Likelihood	0.101	0.025	4.040	0.000
**Regression form:** Lotka	*reference category*			
Pareto	0.086	0.017	4.989	0.000
*Study characteristics*				
**Year of Publication:** 1975 or before	0.141	0.046	3.069	0.002
] 1975; 2000 [	*reference category*			
2000 or after	0.064	0.018	3.653	0.000
**Number of estimates:** 1 only	*-0.02*	*0.08*	*-0.24*	*0.81*
] 1; 10 [	*reference category*			
10 or more	0.064	0.024	2.651	0.008
**Period covered:** 1 year	*0.03*	*0.03*	*0.88*	*0.38*
] 1; 50 [	*reference category*			
50 years or more	*0.04*	*0.02*	*1.94*	*0.05*
**Number of countries:** 1 only	*0.03*	*0.02*	*1.22*	*0.23*
] 1; 5 [	*reference category*			
5 or more	-0.101	0.024	-4.206	0.000
*Territorial characteristics*				
**Date of observation:** 1940 or before	-0.105	0.026	-4.086	0.000
] 1940; 2000 [	*reference category*			
2000 or after	*0.00*	*0.02*	*-0.11*	*0.91*
**Total Population:** 10 million or less	-	-	-	-
] 10M; 100M [	*reference category*			
100 million or more	-	-	-	-
Missing data	0.067	0.027	2.512	0.012
**GDP per Capita:** 1000 Current USD or less	-	-	-	-
] 1,000; 10,000 [	*reference category*			
10,000 or more	-	-	-	-
Missing data	*0.01*	*0.02*	*0.66*	*0.51*
**Age of urbanisation:** Old	*0.10*	*0.08*	*1.37*	*0.17*
Recent	0.201	0.077	2.626	0.009
Not applicable	*reference category*			
**Urbanisation rate:** 20% or less	0.137	0.034	4.046	0.000
] 20; 60 [	*reference category*			
60% or more	0.084	0.016	5.243	0.000
Missing data	-	-	-	-

NB. Italic numbers are for non-significant coefficient with a threshold of 5%.

“-” indicates that the category is undefined.

The fixed-study effect model is applied to technical variables only and thus aims at estimating the percentage of variance which is unnecessary because it results only from implementation choices. I find that this share is very important as the *R*^2^ for this model amounts to 39.7%. The main variable in this model is the city definition, although estimation methods and the regression form keep their impact, along with the size of the study (Table 5).

**Table 5:** Fixed effects meta-analysis. Fixed-study effect model regressing *α* value with estimation and study variables.

	Coefficient	Std. error	t-value
Intercept	0.768	0.082	9.372
*Estimation characteristics*			
**City Definition:** LocalUnit	*reference category*		
MorphoCity	0.196	0.026	7.523
MetroArea	0.153	0.039	3.881
VariaMixed	*0.01*	*0.07*	*0.21*
**Population cutoff:** 10,000 or lower	*0.00*	*0.02*	*0.11*
] 10,000; 100,000 [	*reference category*		
100,000 or higher	-	-	-
Missing specification	*0.03*	*0.03*	*0.95*
**Number of Cities:** 30 or lower	*0.01*	*0.01*	*0.71*
] 30; 300 [	*reference category*		
300 or higher	-	-	-
Missing specification	0.051	0.037	1.371
**Estimation Method:** OLS	*reference category*		
Gabaix & Ibragimov	-0.069	0.039	-1.781
Markov Chains	*0.00*	*0.10*	*-0.05*
Maximum Likelihood	*0.00*	*0.03*	*-0.12*
**Regression form:** Lotka	*reference category*		
Pareto	0.091	0.047	1.936
*Study characteristics*			
**Year of Publication:** 1975 or before	*0.13*	*0.10*	*1.23*
] 1975; 2000 [	*reference category*		
2000 or after	*0.05*	*0.05*	*0.93*
**Number of estimates:** 1 only	*-0.05*	*0.12*	*-0.41*
] 1; 10 [	*reference category*		
10 or more	0.083	0.051	1.621
**Period covered:** 1 year	*-0.01*	*0.08*	*-0/16*
] 1; 50 [	*reference category*		
50 years or more	*-0.01*	*0.05*	*-0.14*
**Number of countries:** 1 only	*0.05*	*0.06*	*0.88*
] 1; 5 [	*reference category*		
5 or more	- *-0.04*	*0.07*	*-0.60*

NB. Italic numbers are for non-significant coefficient with a threshold of 5%.

“-” indicates that the category is undefined.

This observation is confirmed and reinforced by meta-analyses disaggregated at the country level. I selected countries with more than 50 estimations drawn from more than 10 different studies (thus excluding the 61 estimations for Morocco, which come from only 7 studies). This corresponds to five national cases: Brazil, India, France, China and the United States (Table 6). When I control from the different base value of *α* in these countries, I find that the explaining power of the models rise significantly. In particular, estimation variables play an outstanding role in Brazil (80% of variations are accounted for by definition and estimation choices only), and to a lesser extent in France (*R*^2^ = 45%) and China (53%). On average, the characteristics of the study alone explain twice less variation in *α*, expect in the US where the models are systematically worse. However in this country, where the estimation was published (i.e. the publication type and content) is relatively more important in predicting the value reported! Finally, except for China, territorial variables have the lowest explaining power of the three sets of variables. Only in China (and maybe Brazil) does the hierarchy of city sizes seem to be influenced by territorial characteristics and so, possibly, to policy interventions. The United States (and India) represent the opposite situation.

**Table 6:** Static OLS meta-analysis by country.

	Brazil	India	France	China	USA
Mean alpha reported	0.962	0.973	0.908	0.898	1.118
Number of estimations	50	61	97	130	252
Number of studies	10	13	16	13	30
*R*^2^ with estimation var. only (%)	80.4	45.3	60.0	52.5	18.1
*R*^2^ with study var. only (%)	56.1	20.4	38.2	12.3	14.9
*R*^2^ with territorial var. only (%)	29.0	11.8	16.8	47.7	3.2
*R*^2^ of the complete model (%)	95.2	67.0	65.9	82.8	36.0
*Effect of city definition (compared to local areas)*					
MorphoCity	−	+	+	+	+
MetroArea	+		+		
VariaMixed	−				
*Effect of population cutoff (compared to a cutoff of 10,000 to 100,000)*					
Low cutoff	+	−			−
*Effect of sample size (compared to a sample of 30 to 300 cities)*					
Small sample	+			−	
*Effect of regression form (compared to the Lotka form)*					
Pareto	−			+	
*Effect of the study’s publication year (compared to 1975 to 2000)*					
After 2000	−	+			
*Effect of the study’s temporal coverage (compared to 2 to 50 years)*					
Less than 2 years				+	
More than 50 years	+			+	+
*Effect of the study’s territorial coverage (compared to over 5 countries)*					
Single territory	+				+
2 to 4 countries					−
*Effect of the study’s number of estimations (compared to more than 10)*					
2 to 10	−				
*Effect of the date (compared to before 1940)*					
2000-2015			+	−	
*Effect of the country’s share of urban population (compared to over 60%)*					
20 to 60%		−		−	

NB. Only the variables with significant coefficients in the complete model are shown in the table. All results can be reproduced online https://clementinegeo.shinyapps.io/MetaZipf/

**Fig 5:**
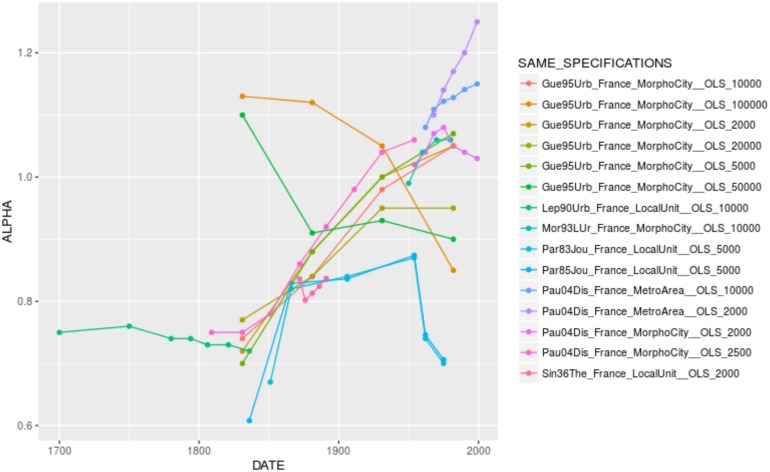
Trajectories of alpha in France by unique specification.

**Fig 6:**
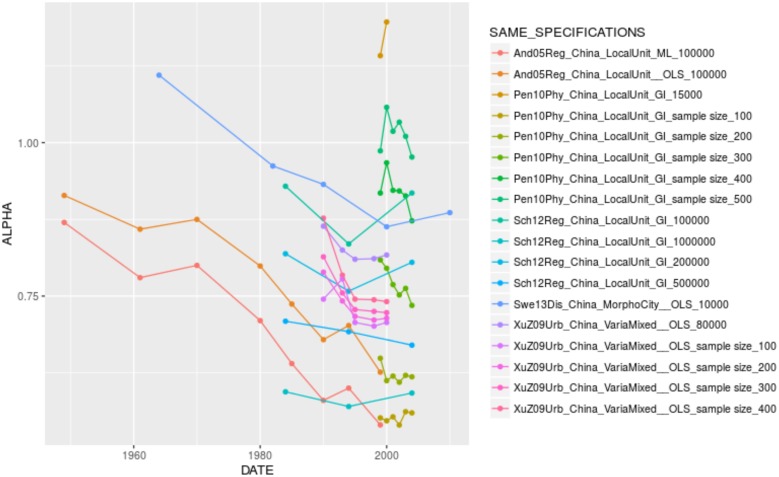
Trajectories of alpha in Brazil by unique specification.

**Fig 7:**
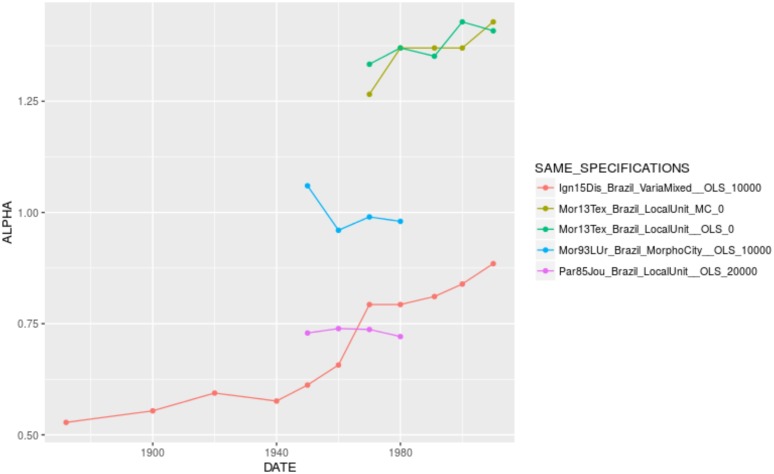
Trajectories of alpha in China by unique specification.

**Fig 8:**
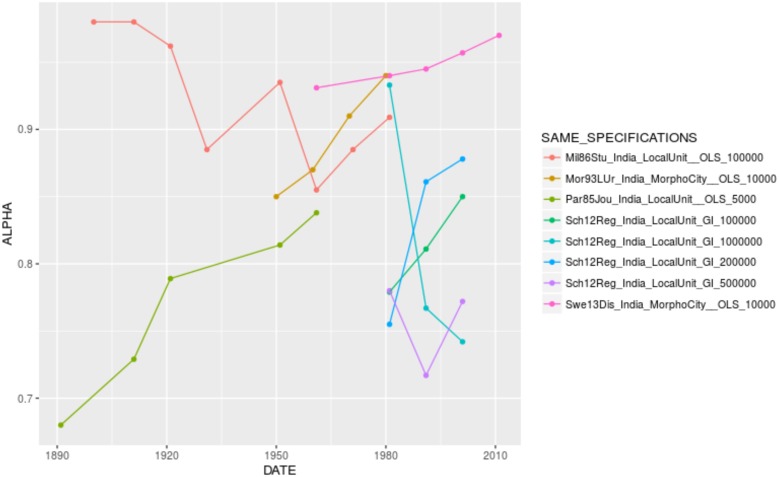
Trajectories of alpha in India by unique specification.

**Fig 9:**
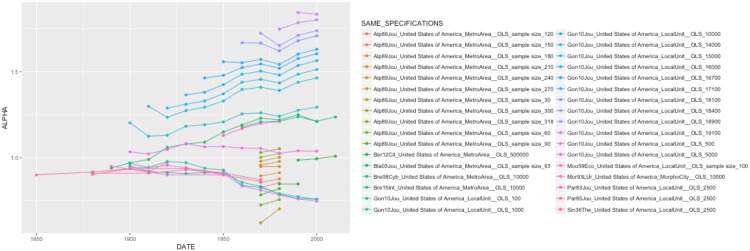
Trajectories of alpha in the USA by unique specification.

When looking for the significant variables playing in the complete models by country, one confirms the effect of city definition and population cutoff, as well as the U-shape relationship between urban hierarchy and the urban transition. It is interesting to see that some specification variables play in reverse, especially in Brazil. In the case of the effect of time, there are opposite coefficients between France (where city size inequality has increased over time, despite the differences in value reported by different authors, Fig 5. The same applies to Brazil, Fig 6) and China (where city size inequality has decreased over time, Fig 7). In other contexts, such as India and the United States, the temporal variation of *α* seems to depend on the specification chosen (Figs 8 and 9).

Having estimated that 40% of variation on average (and up to 80% in Brazil) in *α* are due to technical specifications only, I control for these with a panel model and focus precisely on the spatio-temporal drivers of variations in the next part.

### Dynamic models of urban hierarchy

The random-effect panel model using time-dependent territorial variables confirms my previous findings that the value of *α* is statistically correlated to urbanisation attributes rather than economic and demographic variables. Indeed, with an *R*^2^ of 45.7%, this model indicates that everything else being equal, the inequality coefficient of urban systems in highly urbanised countries is 0.029 point higher than systems where between 20 and 60% of the population is urban. On the contrary, the inequality coefficient of urban systems in highly urbanised countries is 0.091 point higher than systems systems where between 20 and 60% of the population is urban. I thus confirm the U-shaped relationship between the urban transition and city size unevenness.

Finally, a fixed effects panel model of average annual growth rates of Zipf’s coefficients shows only one statistically significant relation: unevenness in city size increases more where it started low (i.e. *α* ≤ 0.75 at time *t*_1_), which is rather trivial as it is where it has more potential for growth. The model is based on 313 longitudinal observations and “explains” 16.2% of variance. However, deceptively, no territorial variable, static or dynamic, is associated with this evolution of urban inequality.

I interpret these dynamic models with care in terms of policy implication, but suggest that leverage for policy makers (if any) would lie in the control of the rural-urban migration rather than natural growth and economic development. However, the models show a high regularity in the value of *α* and potential randomness in its variations.

## Discussion

The last set of dynamic models shows that there is a very large part of evolution left unexplained by the characteristics measured and included in the model (83% to be precise). This could be the effect of rebalancing policies, which would appear in the form of negative residuals (overestimated growth of *α*) over the course and just after such policies in country applying them. The expected countries in this categories should therefore be:

- the Soviet Union in the 1920s when the Desurbanists promoted territorial inequality and the reduction of large cities’ growth.
- the United Kingdom after 1940, when the Barlow commission assigned the task for public policies to rebalance the spatial economy from London and the South East to the Northern cities.
- France after 1960, when it implemented a policy of ‘métropoles d’équilibre’ or balancing metropolises. The government then fostered the development of universities, tribunals and other metropolitan functions in the second-tier cities.
- China after 1978, when Deng Xioaping’s reforms fostered decollectivisation of the agriculture and decentralisation of towns and cities.

None of these appear in the most negative residuals of the fixed-effects panel model of growth rates of Zipf’s coefficient. There is therefore plenty of room for further theory on the drivers of city size unevenness. In particular, generative models need to be implemented to account for different values of rank-size slopes, but also different evolutions of this value. The non-spatial stochastic model of Gibrat is not enough when trying to account for empirical urban systems, which have clear spatial patterns of migrations for example.

## Conclusion

To conclude, there is evidence from this novel meta-analysis that no iron Zipf’s law exists for cities. Furthermore, I found that technical specifications play an unnecessary large role in varying the results and have hampered so far the identification of the territorial drivers of city size inequality.

Using a new and open material for almost 2000 estimations of the rank-size relation, I confirm some of the previous findings, refute the importance of the country size and find that the urban hierarchy is a process that is more linked to the urban process than to economic development. Using residual analysis, I do not find evidence of the effect of planning policy to rebalance city sizes.

As a cumulative contribution to the field, this study comes with open data and a tool for its exploration. The next step in this direction is to look for further spatial mechanisms susceptible to explain the evolution of urbanisation and the urban hierarchy. Without this knowledge, it seems complicated to design effective policies.

## References

[1] Auerbach F. Das Gesetz der Bevölkerungskonzentration. Petermanns Geographische Mitteilungen. 1913;59:74–76.

[2] Zipf GK. Human behavior and the principle of least effort: an introduction to human ecology. Addison-Wesley Press; 1949.

[3] Krugman P. Confronting the mystery of urban hierarchy. Journal of the Japanese and International economies. 1996;10(4):399–418. 10.1006/jjie.1996.0023

[4] Pumain D. Scaling laws and urban systems. SFI Working Paper. 2004;.

[5] Bettencourt LM, Lobo J, Helbing D, Kühnert C, West GB. Growth, innovation, scaling, and the pace of life in cities. Proceedings of the national academy of sciences. 2007;104(17):7301–7306. 10.1073/pnas.0610172104 PMC1852329 17438298

[6] Gomez-Lievano A, Youn H, Bettencourt LM. The statistics of urban scaling and their connection to Zipf’s law. PLoS One. 2012;7(7):e40393 10.1371/journal.pone.0040393 22815745 PMC3399879

[7] Lotka AJ. Elements of physical biology. William & wilkins ed. Baltimore; 1925 Available from: http://agris.fao.org/agris-search/search.do?recordID=US201300526822.

[8] Nitsch V. Zipf zipped. Journal of Urban Economics. 2005;57(1):86–100. 10.1016/j.jue.2004.09.002

[9] Gabaix X, Ibragimov R. Rank- 1/2: a simple way to improve the OLS estimation of tail exponents. Journal of Business & Economic Statistics. 2011;29(1):24–39. 10.1198/jbes.2009.06157

[10] Jiang B, Jia T. Zipf’s law for all the natural cities in the United States: a geospatial perspective. International Journal of Geographical Information Science. 2011;25(8):1269–1281. 10.1080/13658816.2010.510801

[11] Kuninaka H, Matsushita M. Why does Zipf’s law break down in rank-size distribution of cities? Journal of the Physical Society of Japan. 2008;77(11):114801.

[12] Savage SH. Assessing departures from log-normality in the rank-size rule. Journal of archaeological Science. 1997;24(3):233–244. 10.1006/jasc.1996.0106

[13] Moriconi-Ebrard F. L’urbanisation du monde: depuis 1950. Economica; 1993.

[14] Pumain D. Une théorie géographique pour la loi de Zipf. Région et développement. 2012;36:31–54.

[15] Morrill RL. The spatial organization of society. Duxbury Press Belmont; 1970.

[16] Rosen KT, Resnick M. The size distribution of cities: an examination of the Pareto law and primacy. Journal of Urban Economics. 1980;8(2):165–186. 10.1016/0094-1190(80)90043-1

[17] Harris CD. Cities of the Soviet Union: studies in their functions, size, density, and growth. 5 Chicago: Published for Association of American Geographers by Rand McNally; 1970.

[18] Johnson GA. Rank-size convexity and system integration: a view from archaeology. Economic Geography. 1980;56(3):234–247. 10.2307/142715

[19] United Nations. 2015 Revision of World Population Prospects; 2015. Available from: https://esa.un.org/unpd/wpp/.

[20] World Bank. World Bank GDP per capita; 2016. Available from: http://data.worldbank.org/indicator/NY.GDP.PCAP.CD.

[21] United Nations. 2014 Revision of World Urbanization Prospects; 2014. Available from: https://esa.un.org/unpd/wup/.

[22] Soo KT. Zipf’s Law for cities: a cross-country investigation. Regional science and urban Economics. 2005;35(3):239–263. 10.1016/j.regsciurbeco.2004.04.004

[23] Parr JB. A note on the size distribution of cities over time. Journal of Urban Economics. 1985;18(2):199–212. 10.1016/0094-1190(85)90017-8 12313837

[24] González-Val R. The Evolution of U.s. City Size Distribution from a Long-Term Perspective (1900–2000)*. Journal of Regional Science. 2010;50(5):952–972. 10.1111/j.1467-9787.2010.00685.x

[25] Xu Z, Zhu N. City size distribution in China: are large cities dominant? Urban Studies. 2009;46(10):2159–2185. 10.1177/0042098009339432

[26] Cristelli M, Batty M, Pietronero L. There is more than a power law in Zipf. Scientific reports. 2012;2:812 10.1038/srep00812 23139862 PMC3492871

[27] Kuznets S. Economic growth and income inequality. The American economic review. 1955; p. 1–28.

[28] Martinez-Fernandez C, Audirac I, Fol S, Cunningham-Sabot E. Shrinking cities: Urban challenges of globalization. International Journal of Urban and Regional Research. 2012;36(2):213–225. 10.1111/j.1468-2427.2011.01092.x 22518881

